# Effectiveness of a community-based participatory health promotion intervention to address knowledge, attitudes and practices related to intimate partner violence: a quasi-experimental study

**DOI:** 10.1186/s12889-024-18893-0

**Published:** 2024-05-27

**Authors:** Haizana Parween Reyal, Manuja Niranshi Perera, G. N. Duminda Guruge

**Affiliations:** 1 Department of Health Promotion, Faculty of Applied Sciences, Rajarata University of Sri Lanka, Mihintale, Sri Lanka; 2 Department of Public Health, Faculty of Medicine, University of Kelaniya, Ragama, Sri Lanka

**Keywords:** Intimate partner violence, Quasi-experimental study, Knowledge, Attitudes, Determinants, Community-based participatory, Sri Lanka

## Abstract

**Background:**

Intimate partner violence is the most common form of violence experienced by women. It has detrimental consequences. A range of determinants cause intimate partner violence and to reduce it, effective interventions are required to address the determinants. Health promotion interventions have been recommended as effective to enable people to control over the determinants and to improve health. Hence, a community based participatory health promotion intervention was developed and tested in a selected study setting. The objective was to evaluate the effectiveness of a health promotion intervention in terms of addressing knowledge, attitudes and practices related to intimate partner violence.

**Methods:**

A quasi-experimental study was conducted by recruiting ninety women aged 15 to 49 years separately from two health administrative areas identified as the intervention area and the control area from the Kandy district of Sri Lanka. A pretested interviewer-administered questionnaire was used in both pre- and post-assessments. Selected groups of women from the intervention area were facilitated with a health promotion intervention to improve knowledge, attitudes and practices related to intimate partner violence. To evaluate the effectiveness of the intervention descriptive summaries and bivariate analysis were used.

**Results:**

The response rate was 90.9% (*N* = 90) during the pre-assessment and 87.9% (*n* = 87) and 82.8% (*n* = 82) from the intervention and control areas, respectively, during the post-assessment. Statistically significant improvement was reported in the total mean score comprising knowledge, attitudes, practices and identification of determinants from 59.6 to 80.8 in the intervention area [Pre-assessment: Mean = 59.6 (standard deviation-SD) = 17.5; Post-assessment: Mean = 80.8, SD = 19.0; *p* < 0.001) compared to the improvement in the control area from 62.2 to 63.0 (Pre-assessment: Mean = 62.2, SD = 17.3; Post-assessment: Mean = 63.0, SD = 18.9; *p* = 0.654).

**Conclusions:**

The intervention was effective to improve knowledge, attitudes and practices related to intimate partner violence. Hence, the present approach can be used in similar contexts to address the knowledge, attitudes and certain practices related to intimate partner violence.

**Supplementary Information:**

The online version contains supplementary material available at 10.1186/s12889-024-18893-0.

## Background

Worldwide, millions of women suffer from partner violence. The global prevalence of physical and/or sexual intimate partner violence (IPV) among all ever-partnered women was 30.0% [1]. The most recent global prevalence study indicated that 27% of ever-partnered women between 15 and 49 years old have experienced physical and/or sexual IPV, and 13% of women have experienced it during the past year [2]. Although the prevalence rate differs across regions and countries, IPV continues to be a silent global epidemic.

Targeted IPV prevention interventions and responses can reduce IPV [2]. Good practices need to be translated from the descriptive knowledge base to evidence-based with culturally appropriate and acceptable interventions. Although knowledge translation or implementation science has emerged in research, it has been challenging in studies related to IPV [3, 4]. Different models of interventions have been used to address IPV to various degrees. Among these types of diverse responses, community-based approaches such as community mobilization, training of community groups and community-level interventions for families have been reported to reduce IPV [5–7]. Recently, more emphasis has been given to addressing the determinants that increase violence in relationships, and changing community influences and neighboring contexts to prevent IPV [8, 9].

Despite all these recommendations, many countries have not adopted sound mechanisms to address IPV, and the few adopted mechanisms to reduce IPV have not been effectively disseminated [2]. Inadequacy of promising evidence about different approaches remains silent because of scarcity in publishing the findings [10, 11]. In certain studies, the attempts to evaluate the impact of interventions are inadequate [6, 5, 12]. Evaluated approaches also require more testing with a variety of groups in a range of different settings [12, 13]. A review identified that 80% of the evaluations of IPV interventions have emerged from six high-income countries, which comprise only 6% of the global population [10]. Hence, to understand the gaps in evaluating IPV interventions, research needs to be strengthened in low-and middle-income countries. The present study was conducted in Sri Lanka, where IPV has been repeatedly recorded. The prevalence of IPV in Sri Lanka varies between 18.3% and 72%, with the majority reporting IPV between 25 and 35% [14]. A recent study conducted in the selected study setting indicated a high prevalence of IPV with 39.5% physical abuse, 39.0% psychological abuse and 31.3% controlling behaviors [15].

Health promotion is a community-based public health approach that enables people to increase control over the determinants and to improve their health [16]. It encompasses a variety of strategies aimed at improving health outcomes and preventing diseases targetting individuals, communities, or entire populations. They often combined and tailored to the specific context and target population to maximize effectiveness in promoting positive health outcomes [16, 17]. Theories have evolved over the years, demonstrating behavior change. To put forward the theory in to practice, behavior change communication (BCC), information – education and communication (IEC), social change, and empowerment, are some approaches to health promotion [18].

Community-based health promotion represents a conceptual framework emphasizing primary prevention and a population-based perspective emphasizing mobilizing communities to actively participate in achieving program goals; implementing interventions in multiple community settings, using multiple intervention strategies and self-help groups [19]. Furthermore, the community-based participatory research (CBPR) emphasizes partnering with communities and recognizes the importance of involving members of a study population as active and equal participants. CBPR has been identified as an approach that can be used to address health disparities [20]. Considering the range of determinants of IPV and the role of community, the present study tested a community-based participatory health promotion intervention to address knowledge, attitudes and practices related to IPV. Hence, the objective was to evaluate the effectiveness of a health promotion intervention in terms of addressing knowledge, attitudes and practices related to IPV.

## Materials and methods

### Study design and setting

A quasi-experimental pretest–posttest control group design was used. Two local health administrative areas called as Medical Officer of Health (MOH) named Nawalapitiya (intervention area) and Kadugannawa (control area) situated in the Kandy Regional Director of Health Services area were selected. Ethnic composition, socioeconomic backgrounds and the distance between the areas were the reasons to purposively choose these two areas.

### Study population

Ever-married women between the age of 15 to 49 years living in the areas were the study population. Women with diagnosed mental illnesses and cognitive impairments were excluded.

### Sample size and sampling procedure

For the sample size calculation with a significant effect size, clustered sampling and a fixed number of clusters per group determined the difference between the groups with 5% precision and 95% confidence interval (CI) [21]. Intracluster correlation coefficient (ICC) was determined as 0.04 considering similar studies [22, 23].

$$\text{Cluster size}=\frac{\left(1-\uprho \right)}{\left(\frac{{\text{C}}_{1}}{{\text{N}}_{1}}\right)-\uprho }$$

C_1_ = number of clusters in group 1 = 9.

N_1_ = size of group 1 (without adjustment) = 63.

ρ = ICC = 0.04.

The calculated total sample size 180, was further increased by 10% to account for non-response or non-participation errors and the final total sample size was 99 per group [24].

Multistage cluster sampling technique was used to select study participants. As the first stage of sampling, two MOH areas were purposively selected. In the second stage, stratified sampling was used to select three public health midwives (PHM) areas as an urban/semi urban, a rural and an estate area and the same sampling procedure was adapted in the control area to select the PHM areas. Then three villages/streets were selected using simple random technique as clusters from each PHM areas. The cluster was defined as a village/street categorized by the PHM according to the eligible couple registry. Finally, ten participants were selected from the eligible couple registry using simple random sampling.

### Data collection

A pretested interviewer-administered questionnaire titled ‘effectiveness of a health promotion intervention to reduce IPV’ was developed to assess the changes in knowledge, attitudes and practices related to IPV. Trained research assistants collected both pre-assessment and post-assessment data. The data collection of the pre-assessment stage was started in the last week of November, 2017 and the post-assessment data collection was completed in the last week of March, 2019. Participants were given an information sheet while explaining the components of the procedure. Informed written consent was obtained. Privacy and the confidentiality of the participant was safeguarded.

Knowledge on IPV was measured based on receiving awareness on IPV; having the ability to identify different types of IPV in terms of physical, psychological, sexual violence, deprivation, controlling behaviour and economic abuse; having the ability to identify different effects of IPV and awareness on available support services/prevention methods of IPV. Attitudes on IPV were measured and converted into scores based on the participants' own attitudes and the perceptions of other community members’ attitudes towards IPV. Practices on IPV were measured based on the frequency of IPV related behaviours observed in the community as well as occurrence of different types of IPV in their community. Determinants of IPV were measured based on identification of determinants from different levels such as individual, relationship, community and societal level.

### Health promotion intervention

In this community-based participatory approach the participants of the intervention area were facilitated with a health promotion intervention spanned over a period of one year via mother support groups established separately in urban, rural and estate sectors. Initial engagement with the groups were done discussing about “importance of child wellbeing” and “what are the factors affecting child wellbeing?”. As a continuation of this discussion, “relationship between the partners/parents” was identified as a major factor affecting happiness of children. Self-assessing the “satisfaction about the relationship with the partner” supported to initiate the discussion about “violence in intimate relationships”. Then the intervention aimed to improve the understanding about the IPV, diverse determinants that cause IPV and activities and strategies to address selected determinants of IPV. This included components to improve awareness of IPV and change different attitudes and practices that would either support the increase or the continuation of IPV.

The principal investigator conducted group sessions to the mothers’ support groups at households, community centers, child development centers (in estate areas) and clinics. An average of five sessions lasting two to three hours was the expected minimum group exposure. However, the number of members joined and the number of sessions totally conducted varied. Group members conducted activities at individual, household, group and community level as well as disseminated the activities to the community through diverse methods where they were identified as the ‘change agents’ of the community (This health promotion intervention will be published elsewhere).

### Data analysis

Data was initially entered into Microsoft Excel and Statistical Package for the Social Sciences (SPSS) was used to analyze data. For statistical analysis on a logical basis, some independent variables were combined and transformed to reduce the number of categories. A cut-off score was determined by allocating marks for responses of each participant to assess the knowledge, attitudes, practices and identification of determinants from different levels during the pre and post-assessments.

Summary of mean scores on IPV knowledge was calculated based on receiving awareness on IPV (maximum score = 6), having the ability to identify different types of IPV (maximum score = 12), having the ability to identify different effects of IPV (maximum score = 6) and awareness on available support services/prevention methods of IPV (maximum score = 14).

Attitudes measured on a Likert scale, considering the participants' own attitudes and the perceptions of other community members’ attitudes on IPV were converted into scores. The Likert scale used to measure own attitudes were converted to scores on a scale of 0-3 for attitudes favouring IPV (strongly agree–00, agree–0.5, disagree–02, strongly disagree–03, don’t know–00) and attitudes opposing IPV (strongly agree–03, agree–02, disagree–0.5, strongly disagree–00, don’t know–00). The Likert scale used for measuring attitudes of community members converted to scores on a scale of 0-2 (frequent–00, occasional–01, never–02, refused to answer/don’t know–00).

In practices on IPV, the perceived frequency of IPV related behaviours was measured on a scale of always, low, never or don’t know considering IPV victims, perpetrators and incidents. The occurrence of IPV in the community in terms of scolding, slapping or pushing, sexual abuse, deprivation, controlling relationship and economical abuse was measured according to their perception based on frequency (at least once a month, several times a year, at least once a year, almost never, don’t know). IPV occurrence within the village was measured on a scale of 0-9 combining responses for frequency (at least once a month–1, several times a year–2, at least once a year–3, almost never–9, don’t know–0) and prevalence (< 25%–4 marks, 26%–50%–3marks, 51%–75%–2 marks, 76% <–1 mark, don’t know–0 mark).

Determinants of IPV were assessed based on identification of number of determinants as well as the number of determinants that could be identified from different levels such as individual, relationship, community and societal level.

To evaluate the effectiveness of the intervention descriptive summaries and bivariate analysis were used. Responses presented as proportions in the intervention and control groups were compared using Chi Square statistic. When the expected cell count was less than five Fisher’s Exact test was used. When appropriate, within the group comparison was done using MacNemar’s Chi Square statistic. Responses presented as mean scores in the intervention and control groups were compared using the T-test. Pre- and post-assessment mean scores were compared between the groups using Student’s T-test for independent samples and comparison within the groups was done using Paired T-test. One-way ANOVA was done to analyze mean scores with the categories of independent variables. Significance level was set at < 0.05.

## Results

### Socio-demographic characteristics of the study sample

One hundred and eighty women were recruited for this study. The response rate was 90.9% (*n* = 90) from both intervention and control areas during the pre-assessment. The response rate was 87.9% (*n* = 87) for intervention area participants (IAP) and 82.8% (*n* = 82) for control area participants (CAP) during the post-assessment. The socio-demographic characteristics of the study sample during the pre-assessment is presented in Table 1.

**Table 1:** Socio-demographic characteristics of the study sample during the pre-assessment

Characteristics	IAP n (%) (*N* = 90)	CAP n (%) (*N* = 90)	Chi square df *p* value
**Sector of residence**			
Urban	30 (33.3)	30 (33.3)	*X*^2^ = 0.0 df = 2 *p* = 1.0
Rural	30 (33.3)	30 (33.3)	
Estate	30 (33.3)	30 (33.3)	
**Age category**			
15 – 19	1 (1.1)	4 (4.4)	*X*^2^ = 11.6 df = 3 *p* = 0.006^*^
20 – 29	37 (41.1)	35 (38.9)	
30 – 39	47 (52.2)	33 (36.7)	
40 – 49	5 (5.6)	18 (20.0)	
**Marital status**			
Married	89 (98.9)	89 (98.9)	*X*^2^ = 1.87 df = 2 *p* = 1.0^*^
Divorced/Separated	1 (1.1)	0 (0.0)	
Widowed	0 (0.0)	1 (1.1)	
**Educational status**			
No schooling	2 (2.2)	6 (6.7)	*X*^2^ = 8.47 df = 6 *p* = 0.201^*^
Grade 1 – 8	13 (14.4)	15 (16.7)	
Grade 9 – G.C.E. (O/L)	29 (32.2)	17 (18.9)	
Passed G.C.E. O/L	20 (22.2)	18 (20.0)	
G.C.E. (A/L)	5 (5.6)	4 (4.4)	
Passed G.C.E. A/L	20 (22.2)	25 (27.8)	
Diploma/degree/higher degree	1 (1.1)	5 (5.6)	
**Household income category**			
Less than Rs. 20,000	20 (22.2)	26 (28.9)	*X*^2^ = 5.67 df = 5 *p* = 0.334^*^
Rs.20,001 – 34,999	28 (31.1)	30 (33.3)	
Rs.35,000 – 49,999	21 (23.3)	16 (17.8)	
Rs.50,000 – 74,999	16 (17.8)	13 (14.4)	
Rs.75,000 ≤	5 (5.5.)	2(2.2)	
Don’t know/refused/no answer	0 (0.0)	3 (3.3)	
**Employment status**			
Housewives	75 (83.3)	65 (72.2)	*X*^2^ = 3.21 df = 1 *p* = 0.073
Employed/Self employed	15 (16.7)	25 (27.8)	

*Abbreviations*: *G.C.E* General Certificate of Examination, *O/L* Ordinary level, *A/L* Advanced level, *Rs.* Sri Lankan rupees

^*^Fisher’s exact test was used

Only three were dropout from the pre-assessment to the post-assessment from the IAP and eight have dropout from the CAP. Among the IAP drop outs, all were married, belonging to the 30 – 39 age category, belonging to diverse educational backgrounds and income levels.

### Changes in knowledge on IPV

Changes in knowledge on IPV was assessed based on receiving awareness on IPV; having the ability to identify different types of IPV; having the ability to identify different effects of IPV and awareness on available support services/prevention methods of IPV. Comparison of proportions in receiving awareness of IPV from different sources in the IAP and CAP in the pre- and post-assessments and the comparison of knowledge on types of IPV, effects of IPV and awareness on support services/prevention methods of IPV in IAP and CAP in the pre and post-assessment are given in the Additional file 1.

Comparison of summary mean total scores on IPV knowledge in the IAP and CAP in the pre- and post-assessments are given in the Table 2.

**Table 2:** Comparison of summary mean total scores on IPV knowledge in the IAP and CAP in the pre- and post-assessment

Component of knowledge	IAP Mean (SD)		CAP Mean (SD)		*p* value (between groups)^**^
	**Pre (*N* = 90)**	**Post (*N* = 87)**	**Pre (*N* = 90)**	**Post (*N* = 82)**	
	*p* value (within IAP)^*^		*p* value (within CAP)^*^		
Have received awareness on IPV (Maximum score = 6)	1.8 (0.7)	2.0 (0.9)	1.7 (0.7)	1.7 (0.7)	pre *p* = 0.108
	*p* = 0.70		*p* = 0.396		post *p* = 0.006
Having the ability to identify different types of IPV (Maximum score = 12)	7.7 (2.9)	10.2 (2.2)	8.0 (2.8)	8.4 (2.7)	pre *p* = 0.533
	*p* < 0.001		*p* = 0.178		post *p* < 0.001
Having the ability to identify different effects of IPV (Maximum score = 6)	1.3 (0.6)	2.4 (1.0)	1.2 (0.6)	1.3 (0.6)	pre *p* = 0.711
	*p* < 0.001		*p* = 1.0		post *p* < 0.001
Awareness on available support services/prevention methods of IPV (Maximum score = 17)	1.7 (1.3)	3.3 (1.3)	1.6 (1.3)	1.9 (1.3)	pre *p* = 0.596
	*p* < 0.001		*p* = 0.066		post *p* < 0.001
Total knowledge score (Maximum score = 41)	12.5 (3.3)	17.9 (3.5)	12.6 (3.3)	13.2 (3.0)	pre *p* = 0.955
	*p* < 0.001		*p* = 0.075		post *p* < 0.001

^*^Paired t-test

^**^Student’s independent sample t-test

### Changes in attitudes on IPV

Comparison of mean scores of attitudes of participants on IPV in the IAP and CAP in the pre- and post-assessments are presented in Table 3.

**Table 3:** Mean scores of perceived attitudes of participants on IPV in the IAP and CAP in the pre- and post-assessments

Type of attitude	IAP Mean (SD)^a^		CAP Mean (SD)^a^		*p* value between groups^**^
	**Pre (*N* = 90)**	**Post (*N* = 87)**	**Pre (*N* = 90)**	**Post (*N* = 82)**	
	*p* value (within IAP)^*^		*p* value (within CAP)^*^		
Physical violence is acceptable when the wife has an irresponsible behaviour	0.5 (0.8)	1.5 (1.0)	0.6 (0.9)	0.9 (0.9)	pre *p* = 0.272 post *p* < 0.001
	*p* < 0.001		*p* = 0.014		
Physical violence is acceptable when the husband has consumed alcohol	1.4 (1.0)	2.6 (0.5)	1.4 (1.0)	1.8 (1.0)	pre *p* = 0.529 post *p* < 0.001
	*p* < 0.001		*p* < 0.001		
Physical violence is acceptable when the husband has uncontrolled anger	1.0 (0.9)	2.2 (0.8)	1.1 (1.0)	1.4 (1.0)	pre *p* = 0.372 post *p* < 0.001
	*p* < 0.001		*p* = 0.023		
The attention given by the community members towards the violent incident depends on the harm it has caused	0.5 (0.8)	0.8 (1.1)	0.7 (1.0)	0.8 (1.0)	pre *p* = 0.125 post *p* = 0.947
	*p* = 0.002		*p* = 0.025		
Violence between intimate partners is influenced by the media	1.6 (1.1)	2.6 (0.7)	1.7 (1.1)	1.6 (1.2)	pre *p* = 0.974 post *p* < 0.001
	*p* < 0.001		*p* = 0.320		
Violence between intimate partners is influenced by the social media	1.9 (1.2)	2.7 (0.6)	2.1 (1.2)	2.2 (1.1)	pre *p* = 0.354 post *p* < 0.001
	*p* < 0.001		*p* = 0.481		
Violence between intimate partners is influenced by the interferences of friends and family members	2.1 (1.0)	2.5 (0.9)	2.1 (1.1)	2.1 (1.1)	pre *p* = 0.678 post *p* = 0.011
	*p* < 0.001		*p* = 0.887		
Violence between intimate partners cannot be prevented	0.7 (0.9)	2.0 (0.8)	0.7 (1.0)	0.8 (1.0)	pre *p* = 0.780 post *p* < 0.001
	*p* < 0.001		*p* = 0.107		
Less cohesiveness with the community increases IPV	1.6 (1.1)	1.7 (1.1)	1.5 (1.2)	1.4 (1.1)	pre *p* = 0.350 post *p* = 0.167
	*p* = 0.746		*p* = 0.444		

^*^Paired t-test

^**^Student’s independent sample t-test

^a^Maximum score = 3

Comparison of mean scores of perceived attitudes of community members on intimate partner physical violence in the IAP and CAP in the pre- and post-assessments are presented in Table 4.

**Table 4:** Mean scores of perceived attitudes of community members on intimate partner physical violence in the IAP and CAP in the pre- and post-assessments

Type of attitude	IAP Mean^a^ (SD)		CAP Mean^a^ (SD)		*p* value between groups^**^
	**Pre (*N* = 90)**	**Post (*N* = 87)**	**Pre (*N* = 90)**	**Post (*N* = 82)**	
	*p* value (within IAP)^*^		*p* value (within CAP)^*^		
If the wound resulting from a beating of the husband is small there is nothing much to worry about that	0.6 (0.7)	0.9 (1.0)	0.4 (0.7)	0.5 (0.8)	pre *p* = 0.183 post *p* = 0.005
	*p* = 0.001		*p* = 0.302		
Violence should be tolerated in an intimate relationship	0.3 (0.6)	0.6 (0.6)	0.2 (0.4)	0.3 (0.6)	pre *p* = 0.225 post *p* < 0.001
	*p* < 0.001		*p* = 0.062		
As a woman the wife should tolerate violence	0.3 (0.6)	0.7 (0.7)	0.2 (0.5)	0.2 (0.6)	pre *p* = 0.447 post *p* < 0.001
	*p* < 0.001		*p* = 0.572		
The wife should tolerate husbands behaviour for the sake of family wellbeing	0.1 (0.3)	0.3 (0.4)	0.1 (0.4)	0.2 (0.6)	pre *p* = 0.516 post *p* = 0.057
	*p* < 0.001		*p* = 0.191		
It is acceptable to scold the wife, rather than beating	0.7 (0.8)	1.3 (0.8)	0.6 (0.9)	0.8 (1.0)	pre *p* = 0.298 post *p* < 0.001
	*p* < 0.001		*p* = 0.051		
Outsiders should not intervene to solve IPV because it is a personal matter	0.2 (0.5)	0.3 (0.5)	0.1 (0.4)	0.3 (0.7)	pre *p* = 0.154 post *p* = 0.873
	*p* = 0.020		*p* = 0.005		
IPV would be resolved automatically with time	0.2 (0.6)	0.7 (0.8)	0.2 (0.5)	0.2 (0.5)	pre *p* = 0.633 post *p* < 0.001
	*p* < 0.001		*p* = 1.0		

^*^Paired t-test

^**^Student’s independent sample t-test

^a^Maximum score = 2

### Changes in practices related to IPV

Practices related to IPV are presented based on the perceived IPV related practices observed among the villagers and perceived frequency of occurrence of IPV in the community. Comparison of mean scores of observed practices of IPV in the IAP and CAP in the pre- and post-assessments and the comparison of mean scores of frequency of IPV practices in the IAP and CAP in the pre- and post-assessments are presented in the Additional file 2.

Statistically no significant change observed for frequency of perceived different types of IPV practices in the community during pre-assessment and post-assessment between groups in any type of IPV. Comparison of summary mean total scores of IPV practices between IAP and CAP in the pre- and post-assessments are presented in Table 5.

**Table 5:** Comparison of summary mean total scores of IPV practices between IAP and CAP in the pre - and post-assessments

Practices of IPV	IAP Mean (SD)		CAP Mean (SD)		*p* value between groups^**^
	**Pre (*N* = 90)**	**Post (*N* = 87)**	**Pre (*N* = 90)**	**Post (*N* = 82)**	
	*p* value (within IAP)^*^		*p* value (within CAP)^*^		
Observed practices of IPV (Maximum score = 8)	2.7 (2.4)	4.8 (1.9)	3.3 (2.3)	3.5 (2.2)	pre *p* = 0.089 post *p* < 0.001
	*p* < 0.001		*p* = 0.425		
Occurrence of IPV (Maximum score = 54)	29 (14.9)	29.4 (13.0)	31.3 (15.3)	28.7 (16.7)	pre *p* = 0.380 post *p* = 0.783
	*p* = 0.809		*p* = 0.169		
Total of practices score (Maximum score = 62)	31.7 (15.8)	34.2 (13.8)	34.6 (15.9)	32.2 (17.6)	pre *p* = 0.277 post *p* = 0.424
	*p* = 0.124		*p* = 0.211		

^*^Paired t-test

^**^Student’s independent sample t-test

### Identification of determinants of IPV

Additional file 3 presents identification of different levels of determinants of IPV in IAP and CAP in the pre- and post-assessments. There was no statistically significant difference between the groups in identifying different levels of determinants of IPV during pre-assessment. However, there was a statistically significant difference between the groups in identifying different levels of determinants of IPV in individual (*p* < 0.001) and relationship (*p* < 0.001) levels during post-assessment.

### Overall change in knowledge, attitudes, practices and identification of determinants of IPV

Comparison of total mean scores of knowledge, attitudes, practices and determinants of IPV in IAP and CAP in the pre- and post-assessments presented in Table 6.

**Table 6:** Comparison of total mean scores of knowledge, attitudes, practices and determinants of IPV in IAP and CAP in the pre - and post-assessments

Component	IAP Mean (SD)		CAP Mean (SD)		*p* value between groups^**^
	**Pre (*N* = 90)**	**Post (*N* = 87)**	**Pre (*N* = 90)**	**Post (*N* = 82)**	
	*p* value (within IAP)^*^		*p* value (within CAP)^*^		
Knowledge score (Maximum score = 41)	12.6 (3.4)	17.9 (3.5)	12.7 (3.1)	13.2 (3.0)	pre *p* = 0.955 post *p* < 0.001
	*p* < 0.001		*p* = 0.075		
Attitude score (Maximum score = 41)	14.5 (4.9)	25.1 (5.0)	14.2 (4.7)	15.6 (4.7)	pre *p* = 0.546 post *p* < 0.001
	*p* < 0.001		*p* = 0.002		
Practice score (Maximum score = 62)	31.7 (15.8)	34.2 (13.8)	34.6 (15.9)	32.2 (17.6)	pre *p* = 0.277 post *p* = 0.424
	*p* = 0.124		*p* = 0.211		
Determinants score (Maximum score = 6)	0.8 (0.8)	3.6 (1.5)	0.7 (0.7)	2.0 (1.0)	pre *p* = 0.323 post *p* < 0.001
	*p* < 0.001		*p* < 0.001		
Total score (Maximum score = 150)	59.6 (17.5)	80.8 (19.0)	62.2 (17.3)	63.0 (18.9)	pre *p* = 0.444 post *p* < 0.001
	*p* < 0.001		*p* = 0.654		

^*^Paired t-test

^**^Student’s independent sample t-test

The comparison of the summary of mean scores on IPV knowledge in the IAP and CAP in the pre- and post-assessments shows that the total mean score of IAP (M = 17.9, SD = 3.5) was higher than that of the CAP (M = 13.2, SD 3.0) for the knowledge component during the post-assessment. There was a statistically significant difference between groups in the total knowledge score between groups (*p* < 0.001).

The comparison of summary mean scores on IPV attitudes in the IAP and CAP in the pre-and post-assessments shows that there was no significant difference identified between the two groups' attitudes on IPV (*p* = 0.546) during the pre-assessment. However, IAP reported a total mean score of 25.1 (SD = 5.0), and CAP reported a total mean score of 15.6 (SD = 4.7), with a significant difference between the two groups (*p* < 0.001) during the post-assessment.

The comparison of summary mean scores of IPV practices between IAP and CAP in the pre- and post-assessments shows that there was no statistically significant difference in total mean scores for IPV practices between groups during the pre-assessment (*p* = 0.277) and the post-assessment (*p* = 0.424).

The comparison of summary mean scores of identifying determinants in IAP increased to 3.6 (SD = 1.5) from 0.8 (SD = 0.8) in the intervention area, with a statistically significant difference (*p* < 0.001) during the post-assessment.

## Socio-demographic characteristics of the IAP with the overall outcome of the intervention

Total scores revealed statistically significant improvements in the IAP during the post-assessment. Hence, the post-assessment data of the IAP was further analyzed based on the sector differences and other socio-demographic characteristics. Additional file 4 present the socio-demographic characteristics of the IAP with the overall outcome of the intervention. The improvement did not reveal any statistical significant difference between the categories of sector of residence, age, marital status, levels of education, income or employment status of participants.

The comparison of the total score of IPV computed with mean scores of ‘knowledge on IPV’, ‘attitudes on IPV’, ‘practices of IPV’ and ‘identification of determinants of IPV’ (IPV total score) among sector of residence (urban, rural and estate) during pre and post-assessment. Pre-assessment reported significant differences between sectors only in attitudes score (*p* = 0.008) and total score (*p* = 0.011) where the urban sector had the highest score (M = 64.1, SD = 20.5) and the estate sector had the lowest score (M = 51.9, SD = 13.6). However, in the post-assessment total scores did not have a significant difference between the sectors (*p* = 0.260) where all sectors have shown an improvement in the total mean (urban M = 83.9, SD = 23.4; rural M = 82.4, SD = 17.0; estate M = 76.0, SD = 15.7). There was a statistically significant difference within all sectors (*p* < 0.001) when comparing the pre- and post-assessments.

## Discussion

The study aimed to evaluate the effectiveness of a community-based participatory health promotion intervention in terms of addressing knowledge, attitudes and practices related to IPV. The main findings reveal that the intervention was effective to improve knowledge, attitudes and practices related to IPV. The changes would have resulted due to the influences within the mothers support groups as well as due to the actions that were targeted on identified determinants of IPV from different levels.

The community involvement for health promotion can be represented in many ways [17]. In the present community-based participatory health promotion intervention, involving mother support groups would have influenced shared experiences, peer support, and group dynamics to foster positive changes within the group members [17]. It would have particularly effective in improving the knowledge, attitudes and practices related to IPV.

Changes in knowledge, attitudes and beliefs regarding IPV are some common outcome measures of evaluating the effectiveness of an IPV prevention program. Attempts focused on communities have basically tried to identify the relationship between attitude and behavior as a way of responding to IPV [25, 26]. Even commonly used community-based approaches, including interventions targeted at subgroups of the population, are focusing on changing individual attitudes and behavior [27]. However, changes only in knowledge and attitudes cannot adequately predict behavior change [28, 29]. The same was observed from this study, where the intervention was effective in significant positive changes in knowledge, attitudes and but only in certain practices of IPV in the intervention area compared to the control area.

Experimental study designs have identified positive changes in IPV knowledge and attitudes [12]. Although a randomized control trial (RCT) is the recommended study design to test hypotheses, within the natural social setting, it is difficult to conduct an RCT due to the limitations in controlling external factors and interactions between the internal factors. Hence, quasi-experimental designs are recommended and used for assessing the effectiveness of interventions using experimental and control groups [7, 30]. In the study area selection, there was no significant difference between the study groups in their sociodemographic characteristics. Hence, the two study areas selected are appropriate for a comparison of changes in the intervention.

Studies measuring the knowledge, attitudes, and practices of IPV were common among health-related staff for the identification and management of IPV [31–33]. However, studies are inadequate on the implications of knowledge and attitudes of the public to identify and manage IPV in their communities. Although research on the prevalence and health effects of IPV has increased, gaps in knowledge still exist [34]. In this study, awareness about IPV was increased due to the intervention, which shows that deliberate interventions are important to improve IPV awareness. Knowledge on types of abuse, effects and prevention of IPV improved in both IAP and CAP, but the magnitude of improvement was significantly higher in IAP. However, one reason for the low average improvement may be that all the participants of the study sample were not involved with the intervention. However, the statistical significance reveals that the intervention has been disseminated to the nonparticipants.

Attitudes are important in IPV prevention, although the link between attitude and behavior may be weaker at the points of interventions [26]. In this study, the questions on IPV attitudes were developed considering the studies reported in the Sri Lankan context [35–37]. Mean comparison of attitudes on IPV measured on a Likert scale have reflected that the study has been effective in changing the attitudes toward IPV. Attitudes of both villagers and participants were assessed, and IAP reported statistically significant improvement compared to CAP for certain powerful prevailing attitudes. It did not reveal that attitudes that favored IPV were completely reduced among the villagers. However, the comparative frequency of favoring has reduced from ‘frequently’ to ‘occasional’ or ‘agreeing’ to ‘disagreeing’. Adhering to a Likert scale without a midpoint (strongly agree, agree, disagree, strongly disagree and don’t know) avoids neutral answers and provides a strategy for identifying the attitude as supporting or opposing IPV. The questionnaire developed in this study can be improved further to be used in other intervention studies on violence in similar settings. One area of improvement may be forming attitude-related questions in the same directions of agreement with favoring or opposing IPV.

Norms prevailing subordinate status were reflected in studies where the majority of the women had norms justifying the use of violence by male partners [36]. Attitudes of participants for strong disagreements on *‘physical violence is acceptable when the husband has consumed alcohol, when the wife has irresponsible behavior, when the husband has uncontrolled anger’* were significantly changed*.*

Addressing IPV is a long-term process. IPV prevention studies have adopted follow-up assessments at least six months after the intervention to measure behavior changes [7]. This study did not reveal a statistically significant reduction in different types of IPV prevailed in the intervention area in the post-assessment. However, the reduction in the response of ‘almost never’ during the post-assessment may reflect an increased understanding of IPV occurrence within the community. Practices of IPV victims in not revealing IPV due to shame and fear have been reduced in the IAP. This may reflect a conducive atmosphere among women in discussing IPV. The reduction in the number of perpetrators boasting about their behaviors and villagers in glamourizing IPV incidents may be due to the changes dispersed within the villages.

A gap exists in IPV prevention efforts due to the operation of multiple determinants, which requires responses from various stakeholders [12]. Hence, the ability of participants to identify various determinants is important because many of the IPV determinants can be addressed if well understood by people [22]. The present study focused on improving the identification of different levels of determinants that cause IPV.

Attitudes such as individual perception of accepting violence, community norms on rigid gender roles and emphasizing male dominance are prompting IPV [11, 12, 22, 29]. The theory of gender and power provides a theoretical underpinning to understand the relationship between men and women in terms of the gender division of labour, the division of power, and the social norms attached to gender [38]. Inequalities between women and men are one of the ways in which on how gender can influence the distribution of power at different levels of society [39]. The defined set of identities, attitudes and behaviours that societies place as appropriate for women and men cause serious unwelcome consequences from the intimate sphere of the domestic environment to the highest levels of political decision-making [39]. Hence. power must be viewed in both ‘private’ and ‘public’ domains where it can shape outcomes creating rigid hierarchies in the systems starting in household environments where men’s power influences women in everyday life [39]. Hence, the interventions similar to the present study can lead to empowering women within their household environment by having appropriate knowledge and attitudes on IPV.

The sociodemographic characteristics of the IAP did not show any statistically significant relationship with the intervention outcomes. However, as an important main variable of the present study, further analysis of sector residence on the intervention outcome showed that compared to the pre-assessment, all sectors had a significant improvement in all three sectors in the post-assessment. Although there was an improvement, in the post-assessment between the sectors or within the categories of other sociodemographic factors there were no statistically significant differences in the intervention outcome. Hence, it was evident that the present health promotion intervention can be applied to any group of people where it will not be influenced by the sector of residence, levels of education, income or employment status of participants.

Addressing IPV in a community requires more planning and strategies because of the sensitivity of the problem as well as the safety of both the facilitators and the participants [11]. The main principles of this health promotion intervention involve people on day to day life in a community centered mode, while identifying and addressing the determinants with the use of diverse but complementary approaches [16]. In the present study, the groups were engaged in the intervention as a part of their day to day life. They were facilitated in understanding about IPV. The determinants were identified from individual, relationship, community and societal level and analyzed among the groups. Appropriate activities were discussed to address the selected determinants. Health promotion interventions also have a multifaceted process that involves ‘interested’ individuals, groups and communities [40]. Hence, the involved mothers’ groups were supported to adopt their own pace based on the group members’ interest in terms of engaging in the intervention process, choosing the determinants, activities to address the selected determinants and measuring the progress. Although empowerment is crucial in health promotion, the use of only an empowerment approach may not be adequate for involving participants in this kind of generating a process of addressing IPV [41]. Furthermore, this study emphasizes community empowerment involving individuals within their groups acting collectively to gain more control over the determinants of IPV and the wellbeing of their communities [41]. According “ladder of citizen participation” model by Arnstein that describe i) nonparticipation (no power), ii) degrees of tokenism (counterfeit power), and iii) degrees of citizen power (actual power), the present study reflects actual power of participants who had the full control about the participation and continuation [42]. Citizen control was reflected in rural and urban settings where they had the voluntary participation. However, in the estate sectors a partnership mode could be identified where the linked administration had an influence towards the participation [42]. Similarly, the use of other approaches such as the social change approach may also focus on making changes to the physical and social environment to support individuals in making decisions to improve their health and wellbeing rather than improving the knowledge, attitudes and practices of the participants [41].

As a major public health issue driven by powerful social norms, interventions may require more time to reduce IPV behaviours. Hence, the research question of the present study was addressed using only a quantitative method to evaluate the effectiveness of the intervention in terms of changes in the knowledge, attitudes and practices related to IPV. However, the use of a mixed method including a qualitative component would have yielded more details on the group cultures and conditions that would have led to the changes in attitudes and certain practices related to IPV. Hence, not incorporating a mixed method can be considered a limitation of this study. As another limitation, social desirability bias would have influenced and underestimated IPV occurrence [43]. This was more reflected when the groups tended to discuss intimate matters. Hence, discussing IPV occurrence, norms and attitudes was done indirectly to reduce social desirability bias.

## Conclusions

The study revealed that the community-based participatory health promotion intervention was effective in positively changing knowledge and attitudes of IPV and certain IPV practices and improving the understanding of perceived determinants. Hence, as a method of addressing IPV, an organized and systematic mechanism can be used to implement community-based participatory health promotion interventions to improve the knowledge and attitudes of community members regarding IPV.

### Supplementary Information

Supplementary Material 1.

Supplementary Material 2.

Supplementary Material 3.

Supplementary Material 4.

## Data Availability

The data used are available on reasonable request from the corresponding author.
