# Supplementary material for: Effectiveness of a community-based participatory health promotion intervention to address knowledge, attitudes and practices related to intimate partner violence: a quasi-experimental study
Source: BMC Public Health. 2024 May 27;24:1417. doi: 10.1186/s12889-024-18893-0 (PMC11131198; doi:10.1186/s12889-024-18893-0)
Supplement: Supplementary file 1 — Supplementary Material 1. [file 12889_2024_18893_MOESM1_ESM.docx]

**Comparison of proportions in receiving awareness of IPV from different sources in the IAP and CAP in the pre and post assessment**

| **Source of receiving awareness** | **IAP No. (%)** | | **CAP No. (%)** | | **p value**  **(between groups)** |
| --- | --- | --- | --- | --- | --- |
|  | **Pre**  **(N=90)** | **Post**  **(N=87)** | **Pre**  **(N=90)** | **Post**  **(N=82)** |  |
|  | **p value**  **(within IAP)** | | **p value**  **(within CAP)** | |  |
| Formal course | 0 (0.0) | 1 (1.1) | 0 (0.0) | 2 (2.4) | pre  p= NA  post p=0.612***** |
|  | NA | | NA | |  |
| Training/workshop | 4 (4.6) | 2 (2.3) | 4 (4.9) | 4 (4.9) | pre  p= 1.0*****  post p=0.433***** |
|  | p=0.625** | | p=1.0** | |  |
| Media | 41 (45.6) | 41 (47.1) | 41 (45.6) | 34 (41.5) | pre  p= 1.0  post p=0.459 |
|  | p=1.0** | | p=0.454** | |  |
| Social media | 3 (3.4) | 7 (8.0) | 1 (1.1) | 1 (1.2) | pre  p= 0.621*****  post p=0.065***** |
|  | p=0.001 | | p=1.0** | |  |
| Friends | 24 (26.7) | 28 (32.2) | 16 (17.8) | 11 (13.4) | pre p=0.151  post p=0.004 |
|  | p=0.061 | | p=0.289** | |  |
| Other | 40 (44.4) | 60 (69.0) | 36 (40.0) | 47 (57.3) | pre  p= 0.546  post p=0.116 |
|  | p<0.001** | | p<0.001 | |  |
| Total reception of awareness | 87 (96.7) | 87 (100.0) | 85 (94.4) | 80 (97.6) | pre  p=0.720  post p=0.234 |
|  | NA | | p=0.003** | |  |

* Fisher’s Exact test; **MacNemar’s Chi Square test; NA=Not Applicable because constant measurements

**Comparison of knowledge on types of IPV, effects of IPV and awareness on support services/prevention methods of IPV in IAP and CAP in the pre and post assessment**

| **Type of abuse** | **IAP Mean^#^ (SD)** | | **CAP Mean^#^ (SD)** | | **p value between groups**** |
| --- | --- | --- | --- | --- | --- |
|  | **Pre**  **(N=90)** | **Post**  **(N=87)** | **Pre**  **(N=90)** | **Post**  **(N=82)** |  |
|  | **p value**  **(within IAP)*** | | **p value**  **(within CAP)*** | |  |
| **Component of knowledge: Ability to identify different types of IPV** | | | | | |
| Slapping or pushing as violence (Physical) | 1.4 (0.7) | 2.0 (0.2) | 1.4 (0.6) | 1.6 (0.6) | pre  p=0.735  post p<0.001 |
|  | p<0.001 | | p=0.001 | |  |
| Scolding as violence (Psychological) | 1.2 (0.7) | 1.8 (0.5) | 1.2 (0.6) | 1.3 (0.6) | pre  p=1.0  post p<0.001 |
|  | p<0.001 | | p=0.072 | |  |
| Forcing sex as violence (Sexual) | 1.4 (0.7) | 1.9 (0.4) | 1.4 (0.7) | 1.6 (0.7) | pre  p=0.545  post p=0.002 |
|  | p<0.001 | | p=0.01 | |  |
| Deprivation as violence | 1.2 (0.8) | 1.6 (0.7) | 1.3 (0.6) | 1.3 (0.6) | pre  p=0.408  post p=0.035 |
|  | p<0.001 | | p=1.0 | |  |
| Economic abuse as violence | 0.4 (0.5) | 1.5 (0.7) | 0.4 (0.5) | 1.3 (0.6) | pre  p=0.543  post p=0.086 |
|  | p<0.001 | | p<0.001 | |  |
| Controlling behaviour as violence | 1.2 (0.7) | 1.6 (0.6) | 1.3 (0.7) | 1.3 (0.7) | pre  p=0.463  post p=0.007 |
|  | p<0.001 | | p=0.697 | |  |
| (^#^Maximum score=2; *Paired t-test; **Student’s independent sample t-test.) | | | | | |
| **Component of knowledge: Ability to identify different effects of IPV** | | | | | |
| IPV affects health | 31 (34.4) | 53 (60.9) | 26 (31.7) | 31 (37.8) | pre  p=0.752  post p=0.003 |
|  | p<0.001** | | P=0.227** | |  |
| IPV affects economy | 2 (2.2) | 7 (8.0) | 1 (1.1) | 1 (1.2) | pre p=1.000*  post p=0.065* |
|  | p=0.125** | | p=1.000** | |  |
| IPV affects family | 31 (34.4) | 63 (72.4) | 31 (34.4) | 30 (36.6) | pre p=1.000  post p<0.001 |
|  | p<0.001** | | p=0.815** | |  |
| IPV affects children | 46 (51.1) | 77 (88.5) | 50 (55.6) | 41 (50.0) | pre  p= 0.550  post p<0.001 |
|  | p<0.001** | | p=0.118** | |  |
| IPV affects society | 3 (3.3) | 6 (6.9) | 0 (0.0) | 0 (0.0) | pre  p= 0.246*  post p=0.029* |
|  | p=0.508** | | NA | |  |
| Other affects | 2 (2.2) | 1 (1.1) | 1 (1.1) | 0 (0.0) | pre  p= 1.000*  post p=1.000* |
|  | p=1.000** | | NA | |  |
| **Component of knowledge: Awareness on prevention methods/support services** | | | | | |
| Social services | 2 (2.3) | 32 (36.8) | 6 (7.3) | 6 (7.3) | pre p=0.497*  post p<0.001 |
|  | p<0.001 | | p=1.000 | |  |
| Health services | 3 (3.3) | 21 (24.1) | 3 (3.7) | 7 (8.5) | pre p=1.000*  post p=0.007 |
|  | p<0.001 | | p=0.219 | |  |
| Women’s organizations | 5 (5.7) | 3 (3.4) | 6 (7.3) | 4 (4.9) | pre  p= 0.550  post p=0.714* |
|  | p=0.727 | | p=0.625 | |  |
| Legal aid | 12(13.8) | 9 (10.3) | 9 (11.0) | 9 (11.0) | pre  p= 0.823  post p=1.000 |
|  | p=0.629 | | p=1.000 | |  |
| Religious institutions | 0 (0.0) | 1 (1.1) | 0 (0.0) | 0 (0.0) | pre  p=NA  post p=1.000* |
|  | NA | | NA | |  |
| Family members | 6 (6.9) | 26 (29.9) | 3 (3.7) | 15 (18.3) | pre  p= 0.351  post p=0 .079 |
|  | p<0.001 | | p=0.002 | |  |
| Villagers | 11 (12.6) | 38 (43.7) | 9 (11.0) | 10 (12.2) | pre  p=0.816  post p<0.001 |
|  | p<0.001 | | p=1.000 | |  |
| Other sources | 4 (4.6) | 21 (24.1) | 4 (4.9) | 3 (3.7) | pre  p=1.000  post p<0.001 |
|  | p<0.001 | | p=1.000 | |  |
| **Total awareness on prevention** | 41 (45.6) | 78 (89.7) | 43 (47.8) | 42 (51.2) | pre p=0.765 |
|  | p<0.001 | | p=0.481 | | post p<0.001 |
| (*Fisher’s Exact test; **MacNemar’s Chi Square test; NA=Not Applicable because constant measurements.) | | | | | |
