# Supplementary material for: Effectiveness of a community-based participatory health promotion intervention to address knowledge, attitudes and practices related to intimate partner violence: a quasi-experimental study
Source: BMC Public Health. 2024 May 27;24:1417. doi: 10.1186/s12889-024-18893-0 (PMC11131198; doi:10.1186/s12889-024-18893-0)
Supplement: Supplementary file 2 — Supplementary Material 2. [file 12889_2024_18893_MOESM2_ESM.docx]

**Comparison of mean scores of observed practices of IPV in the IAP and CAP in the pre and post assessment**

| **Practices of IPV^#^** | **IAP Mean (SD)** | | **CAP Mean (SD)** | | **p value between groups**** |
| --- | --- | --- | --- | --- | --- |
|  | **Pre**  **(N=90)** | **Post (N=87)** | **Pre**  **(N=90)** | **Post**  **(N=82)** |  |
|  | **p value**  **(within IAP)*** | | **p value**  **(within CAP)*** | |  |
| Victims are ashamed to reveal IPV | 0.5 (0.7) | 1.0 (0.5) | 0.6 (0.7) | 0.5 (0.6) | pre p=0.308  post p<0.001 |
|  | p<0.001 | | p=0.436 | |  |
| Victims are afraid to reveal about IPV | 0.6 (0.7) | 1.0 (0.6) | 0.7 (0.7) | 0.6 (0.7) | pre p=0.446  post p<0.001 |
|  | p<0.001 | | p=0.552 | |  |
| The perpetrator will boast about the IPV acts | 0.9 (0.8) | 1.5 (0.6) | 1.1 (0.8) | 1.2 (0.7) | pre p=0.117  post p=0.045 |
|  | p<0.001 | | p=0.150 | |  |
| Violence incidents are discussed in a glamorized way | 0.7 (0.9) | 1.3 (0.8) | 1.0 (8.6) | 1.1 (0.8) | pre p=0.081  post p=0.069 |
|  | p<0.001 | | p=0.063 | |  |

*Paired t-test; **Student’s independent sample t-test; ^#^Maximum score=2.

**Comparison of mean scores of frequencies of IPV practices in the IAP and CAP in the pre and post assessment**

| **Practices of IPV^#^** | **IAP Mean (SD)** | | **CAP Mean (SD)** | | **p value between groups**** |
| --- | --- | --- | --- | --- | --- |
|  | **Pre**  **(N=90)** | **Post (N=87)** | **Pre**  **(N=90)** | **Post**  **(N=82)** |  |
|  | **p value**  **(within IAP)*** | | **p value**  **(within CAP)*** | |  |
| Slapping or pushing (Physical) | 1.6 (1.2) | 1.6 (1.1) | 1.9 (1.3) | 1.7 (1.2) | pre p=0.065  post p=0.493 |
|  | p=1.000 | | p=0.077 | |  |
| Scolding  (Psychological) | 1.5 (1.1) | 1.6 (1.0) | 1.6 (1.3) | 1.4 (1.2) | pre p=0.529  post p=0.206 |
|  | p=0.235 | | p=0.092 | |  |
| Forcing for sex  (Sexual) | 2.1 (1.9) | 2.2 (1.8) | 2.6 (1.8) | 2.2 (1.9) | pre p=0.129  post p=0.867 |
|  | p=0.832 | | p=0.124 | |  |
| Deprivation | 1.9 (1.6) | 1.8 (1.4) | 1.9 (1.7) | 1.9 (1.7) | pre p=0.855  post p=0.576 |
|  | p=0.582 | | p=1.000 | |  |
| Economic abuse | 1.7 (1.6) | 1.9 (1.5) | 2.1 (1.7) | 1.8 (1.7) | pre p=0.195  post p=0.742 |
|  | p=0.428 | | p=0.172 | |  |
| Controlling behaviour | 2.1 (1.7) | 1.9 (1.7) | 2.1 (1.7) | 1.9 (1.8) | pre p=0.859  post p=0.950 |
|  | p=0.528 | | p=0.320 | |  |

*Paired t-test; **Student’s independent sample t-test; ^#^Maximum score=4.
