# Supplementary material for: Effectiveness of a community-based participatory health promotion intervention to address knowledge, attitudes and practices related to intimate partner violence: a quasi-experimental study
Source: BMC Public Health. 2024 May 27;24:1417. doi: 10.1186/s12889-024-18893-0 (PMC11131198; doi:10.1186/s12889-024-18893-0)
Supplement: Supplementary file 3 — Supplementary Material 3. [file 12889_2024_18893_MOESM3_ESM.docx]

**Identification of different levels of determinants of IPV in IAP and CAP in the pre and post assessments**

| **Level of determinant** | **IAP No. (%)** | | **CAP No. (%)** | | **p value between groups*** |
| --- | --- | --- | --- | --- | --- |
|  | **Pre (N=90)** | **Post (N=87)** | **Pre**  **(N=90)** | **Post**  **(N=82)** |  |
|  | **p value**  **(within IAP)*** | | **p value**  **(within CAP)*** | |  |
| **Individual level** | | | | | |
| Aware of one determinant | 40 (44.4) | 26 (29.9) | 39 (43.3) | 53 (64.6) | pre p=0.413  post p<0.001* |
| Aware of two determinants | 12 (13.3) | 51 (58.6) | 7 (7.8) | 6 (7.3) |  |
| Aware of three determinants | 0 (0.0) | 2 (2.3) | 0 (0.0) | 0 (0.0) |  |
|  | p=0.370* | | p=0.282* | |  |
| **Relationship level** | | | | | |
| Aware of one determinant | 7 (7.8) | 24 (27.6) | 6 (6.7) | 4 (4.9) | pre p=0.773  post p<0.001* |
| Aware of two determinants | 0 (0.0) | 2 (2.3) | 0 (0.0) | 0 (0.0) |  |
|  | p=0.028* | | p=1.000* | |  |
| **Community level** | | | | | |
| Aware of one determinant | 2 (2.2) | 9 (10.3) | 2 (2.2) | 2 (2.4) | pre p=1.000*  post p=0.072* |
| Aware of two determinants | 0 (0.0) | 1 (1.1) | 0 (0.0) | 1 (1.2)) |  |
|  | p=0.876 | | p=1.000* | |  |
| **Societal level** | | | | | |
| Aware of one determinant | 0 (0.0) | 9 (10.3) | 2 (2.2) | 1 (1.2) | pre p=0.497*  post p=0.070* |
| Aware of two determinants | 0 (0.0) | 1 (1.1) | 0 (0.0) | 0 (0.0) |  |
| Aware of three determinants | 0 (0.0) | 1 (1.1) | 0 (0.0) | 0 (0.0) |  |
|  | NA | | p=0.240 | |  |

*Fisher’s Exact test; NA=No statistics are computed because of constant measures.
