# Supplementary material for: Effectiveness of a community-based participatory health promotion intervention to address knowledge, attitudes and practices related to intimate partner violence: a quasi-experimental study
Source: BMC Public Health. 2024 May 27;24:1417. doi: 10.1186/s12889-024-18893-0 (PMC11131198; doi:10.1186/s12889-024-18893-0)
Supplement: Supplementary file 4 — Supplementary Material 4. [file 12889_2024_18893_MOESM4_ESM.docx]

**Socio-demographic characteristics of the IAP with the overall outcome of the intervention**

| **Characteristics** | **n**  **(N=87)** | **Mean** | **p value**  **between groups*** |
| --- | --- | --- | --- |
| **Sector of residence** | | | |
| Urban | 28 | 83.86 | p=0.260 |
| Rural | 30 | 82.38 |  |
| Estate | 29 | 76.09 |  |
| **Age category** | | | |
| 15 – 19 | 1 | 73.00 | p=0.654 |
| 20 – 29 | 37 | 82.12 |  |
| 30 – 39 | 44 | 78.89 |  |
| 40 – 49 | 5 | 88.70 |  |
| **Marital status** | | | |
| Married | 86 | 80.52 | p=0.275 |
| Divorced/Separated | 1 | 101.50 |  |
| Widowed | 0 |  |  |
| **Educational status** | | | |
| No schooling | 2 | 71.75 | p=0.185 |
| Grade 1 – 8 | 12 | 79.25 |  |
| Grade 9 – G.C.E. (O/L) | 29 | 78.71 |  |
| Passed G.C.E. O/L | 19 | 80.08 |  |
| G.C.E. (A/L) | 5 | 65.90 |  |
| Passed G.C.E. A/L | 19 | 90.32 |  |
| Diploma, degree, higher degree | 1 | 82.00 |  |
| **Household income category** | | | |
| <Rs. 20,000 | 20 | 76.07 | p=0.524 |
| Rs.20,001 – 34,999 | 27 | 80.87 |  |
| Rs.35,000 – 49,999 | 21 | 80.29 |  |
| Rs.50,000 – 74,999 | 14 | 84.79 |  |
| Rs.75,000 ≤ | 3 | 81.67 |  |
| Don’t know/refused/no answer | 2 | 101.50 |  |
| **Employment status** | | | |
| Housewives | 73 | 81.39 | p=0.482 |
| Employed/Self employed | 14 | 77.46 |  |

*One-way ANOVA

**Comparison of total mean scores of knowledge, attitude, practices, determinants and total IPV scores among sector of residence during pre and post assessment**

| **Component** | **Urban Sector**  **Mean (SD)** | | **Rural Sector**  **Mean (SD)** | | **Estate Sector**  **Mean (SD)** | | **p value between groups*** |
| --- | --- | --- | --- | --- | --- | --- | --- |
|  | **Pre N=30** | **Post**  **N=28** | **Pre N=30** | **Post N=30** | **Pre N=30** | **Post**  **N=29** |  |
|  | **p value ****  **(within group)** | | **p value****  **(within group)** | | **p value****  **(within group)** | |  |
| Knowledge score  (Max =41) | 12.3 (3.5) | 18.2 (3.7) | 12.9 (2.5) | 19.2 (3.3) | 12.3 (3.9) | 16.4 (3.0) | pre p=0.688  post p=0.006 |
|  | p<0.001 | | p<0.001 | | p<0.001 | |  |
| Attitude score  (Max = 41) | 15.6 (4.5) | 25.6 (4.9) | 15.5 (5.2) | 25.8 (5.2) | 12.3 (4.2) | 23.8 (4.8) | pre p=0.008  post p=0.240 |
|  | p<0.001 | | p<0.001 | | p<0.001 | |  |
| Practice score  (Max = 62) | 35.5 (18.2) | 36.6 (17.5) | 33.4 (15.0) | 33.4 (12.3) | 26.6 (12.2) | 32.6 (11.0) | pre p=0.066  post p=0.519 |
|  | p=0.603 | | p=0.5 | | p=0.029 | |  |
| Determinants score  (Max = 6) | 0.7 (0.6) | 3.5 (1.6) | 0.9 (0.8) | 3.9 (1.8) | 0.9 (1.0) | 3.3 (1.0) | pre p=0.204  post p=0.017 |
|  | p<0.001 | | p<0.001 | | p<0.001 | |  |
| Total score  (Max = 150) | 64.1 (20.5) | 83.9 (23.4) | 62.8  (15.5) | 82.4 (17.0) | 51.9 (13.6) | 76.0 (15.7) | pre p=0.011  post p=0.260 |
|  | p<0.001 | | p<0.001 | | p<0.001 | |  |

*ANOVA;**Paired t-test.
